# AI supported diagnostic innovations for impact in global women’s health

**DOI:** 10.1136/bmj-2025-086009

**Published:** 2025-10-10

**Authors:** Nina Linder, Dinnah Nyirenda, Andreas Mårtensson, Harrison Kaingu, Billy Ngasala, Johan Lundin

**Affiliations:** 1 Global Health and Migration Unit, Department of Women’s and Children’s Health, Uppsala university, Uppsala, Sweden; 2 Institute for Molecular Medicine Finland (FIMM), HiLIFE, University of Helsinki, Helsinki, Finland; 3 Muhimbili University of Health and Allied Sciences (MUHAS), Dar es Salaam, Tanzania; 4 Department of Infectious Diseases, Uppsala University Hospital, Uppsala, Sweden; 5 Kinondo Kwetu Hospital, Kwale County, Kenya; 6 Department of Global Public Health, Karolinska Institutet, Stockholm, Sweden

## Abstract

**Nina Linder and colleagues** examine how artificial intelligence could be applied to diagnostic methods that rely on highly trained experts, such as cytological screening for cervical cancer, enabling implementation even in resource limited settings

Screening and diagnostic methods are essential for secondary prevention, early detection, and appropriate treatment across a range of women’s health conditions. Cervical cancer provides a compelling example of how screening and timely diagnosis can substantially improve outcomes and drastically reduce mortality, while also reflecting how women’s health priorities have historically been underfunded and under-innovated, especially in low and middle income countries (LMICs). The disease is considered preventable through human papillomavirus (HPV) vaccination and various screening modalities, but there has been limited investment in novel diagnostic tools, and inequities in access to evidence based, cost effective screening prevail, particularly in low resource settings.^1^ ^2^ Cervical cancer screening ever in lifetime among women aged 30-49 years, for example, was reported to be 84% in high income countries and 11% in low income countries.^1^

Artificial intelligence (AI) supported tools, such as cytological cervical cancer screening, self-sampling combined with HPV testing, or AI analysis of cervix images, show great promise and have been successfully implemented at scale in research and clinical settings.^3^ ^4^ ^5^ ^6^ Yet their impact in LMICs remains constrained by persistent health system barriers to implementation and scale-up. Uptake of digital technologies is often hindered by systemic barriers including regulatory uncertainty, infrastructure constraints, and competing economic priorities.^7^ ^8^ In parallel, molecular HPV testing capabilities and HPV vaccine coverage remain limited in many LMICs, with approximately one in five eligible girls receiving the full course of preventive immunisation.^9^ ^10^ Given the 10-20 year lag time before HPV vaccination has a measurable impact on cervical cancer rates, scalable and effective screening remains critical for early detection.

AI models are being studied for prediction of maternal pre-eclampsia, postpartum haemorrhage, and sepsis 11 12; AI image analysis methods can aid diagnosis of sexually transmitted and parasitic infections^13^ ^14^; an AI supported approach to breast cancer detection can either reduce radiologists’ workload in mammography workflows^15^ or, in non-specialist healthcare settings, support radiation-free breast thermography for preliminary risk assessment under clinician oversight.^15^ ^16^ Further, smartphone algorithms can screen non-invasively for anaemia,^17^ and AI enabled point-of-care ultrasonography and cardiotocography show promise for expanded access to obstetric diagnostic measures, such as gestational age, fetal presentation, and fetal heart monitoring^18^ ^19^—all with the potential to reduce avoidable morbidity and mortality.

This article focuses on AI based cervical cancer screening, exploring how AI supported diagnostic tools can be better implemented and scaled up in resource limited settings, using cytological screening for cervical cancer and other women’s health conditions as illustrative examples.

## Barriers to implementation and scale-up

Efforts to implement AI supported diagnostic methods in LMICs face multiple interlocking barriers:

*Health system and infrastructure limitations*—Many AI tools rely on high resolution imaging, consistent electricity, and internet access, which are often unavailable or intermittently available in rural and low resource areas. AI based cervical cancer screening requires advanced imaging instruments and access to staining reagents, AI supported breast cancer screening with mammography is dependent on radiography equipment, and many AI applications rely on access to cloud computing resources. In many LMICs, there is a shortage of trained personnel to operate, maintain, and interpret AI supported diagnostic tools, limiting their usability and sustainability.

*Policy and regulatory uncertainty*—Clear regulatory pathways for the approval and oversight of AI based diagnostic tools are often lacking.^20^ Without robust policy frameworks, governments and health systems are hesitant to adopt or scale up these tools. This leads to major delays in implementation, creates uncertainty for developers, and slows down access to potentially lifesaving innovations. AI supported diagnostic methods bring additional regulatory challenges related to data management, transfer, cloud processing, and storage.

*Financing and procurement bottlenecks*—AI diagnostic methods, even when cost effective in the long term, may face short term affordability issues. Procurement systems in LMICs may struggle to evaluate and purchase innovative technologies, particularly if not bundled with necessary infrastructure or training. Digital pathology and ultrasonography surveys, for example, report cost of equipment, maintenance, and competing use of a limited number of machines as major barriers.^7^ ^21^

*Cultural trust and community engagement*—Community uptake of diagnostic services can be limited by stigma (gynaecological screening), mistrust in technology, or lack of understanding regarding the benefits of screening. Engaging communities early in the design and implementation process is critical to building trust. Health system and policy reviews highlight stigma, embarrassment, and low awareness as key barriers to cervical and breast cancer screening in LMICs, reducing screening uptake independent of technology.^22^

*Innovation design mismatch*—Many AI tools are designed in and for high income contexts and may not reflect the clinical needs and realities of LMICs. AI tools are often ill suited to resource limited settings when they require continuous power, remote cloud computing, a steady supply of reagents, or referral to tertiary care. Sometimes, there is also a mismatch between the healthcare level for which the methods are developed and the level where they are most needed. In a recent scoping review on the use of AI supported digital microscopy in primary healthcare laboratories, only 22 of 3403 articles studied diagnostic solutions that cover the entire diagnostic process from beginning to end in primary healthcare, of which only two relate to women’s health—both focusing on cervical cancer screening with cytology.^23^

*Applicability challenges*—Transferring medical AI methods to different contexts is a well recognised challenge.^24^ Models that perform well at the development site often lose accuracy when applied to data from different populations, healthcare settings, or devices. Performance may also deteriorate after seemingly minor changes within a site, such as software upgrades, device replacements, or laboratory reagent and protocol updates.

Overcoming these barriers requires a holistic, context sensitive approach that involves multiple stakeholders, including policy makers, funders, developers, and health workers who work directly with patients. To ensure that diagnostic innovations translate into meaningful improvements in health outcomes, the entire health system needs to be tackled.^25^

## Case example: AI supported cervical cancer screening

**Fig 1:**
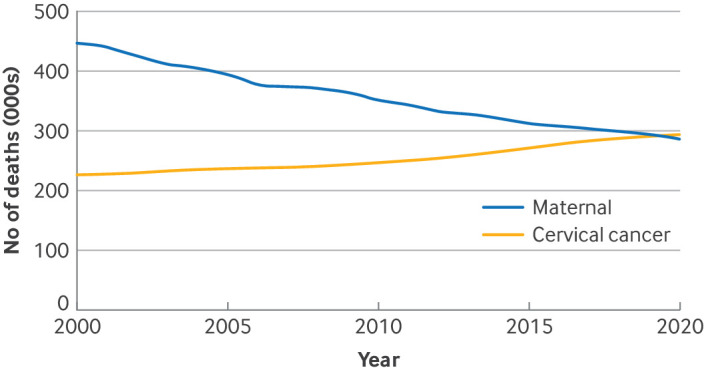
Number of maternal versus cervical cancer deaths globally from 2000 to 2020.^27^ ^28^

Globally, cervical cancer remains the fourth most common cancer in women, with 660 000 new cases in 2022, 94% of which occurred in LMICs.^26^ Notably, in 2020, deaths from cervical cancer surpassed global maternal deaths, underscoring its magnitude as a global women’s health crisis (fig 1).^27^ ^28^ Access to pathology services is limited in most LMICs.^2^ Kenya and Tanzania, for example, have 1.38 and 0.46 pathologists per million inhabitants, respectively.^29^ Thus, cervical cancer is a disease of poverty and inequity.

In many resource limited settings, cervical cancer screening relies on visual inspection with acetic acid, which, despite its immediacy and use by mid-level practitioners, has limited diagnostic accuracy owing to subjectivity and low specificity.^1^ ^30^ Cervical cytology has higher accuracy, but cervical smear services are typically restricted to tertiary healthcare facilities, with delays in result delivery contributing to substantial loss to follow-up.^31^ Testing for HPV—considered a high performance method in cervical cancer screening—is largely inaccessible in resource constrained settings owing to cost, infrastructure, and workforce limitations.^32^ As a result, only about a third of women aged 30-49 globally have ever undergone any cervical cancer screening,^1^ emphasising the urgent need to expand access to effective screening to meet the World Health Organization’s target of screening 70% of women by age 45.^33^

## Case study: AI supported cytological cervical cancer screening in East Africa

Our experience of implementing an AI supported cytological cervical cancer screening system, based on a minimal infrastructure approach, in rural southern Kenya shows both the opportunities and barriers for embedding diagnostic tools in a local health system.^4^ The project involves establishing laboratories with AI diagnostic capability in primary healthcare hospitals, using existing human resources and infrastructure such as electricity, water, and internet connectivity. The method includes staining and digitisation of the cervical smear samples collected by healthcare workers at the primary healthcare hospital. The digital images of the samples are then uploaded via mobile networks to a cloud environment for AI analysis and remote expert verification. The system supports multi-disease diagnostic capabilities making it a scalable and efficient solution for resource limited settings.^34^

To tackle the health system barriers, a dedicated local team was formed, including nurses, laboratory technicians, data managers, pathologists, and clinicians, with clearly defined roles supported by standard operating procedures. All staff members received targeted training in specimen collection, slide preparation, biosafety, digital data entry, and use of the AI tools. We also established a training centre for AI diagnostic tools at the primary study site, where staff from other similar primary healthcare hospitals in Kenya and Tanzania were trained, and the AI method was subsequently transferred to these sites.

Despite attempts to tackle infrastructure barriers, we experienced several challenges. The implementation of cervical smear sample digitisation and AI supported analysis was technically feasible with a turnaround time of approximately 10 to 40 minutes,^4^ but the diagnostic accuracy and capacity was constrained by unreliable reagent supply, inadequate quality of reagents, and power interruptions. Although the AI algorithms were trained with local data, changes in staining reagents from batch to batch often caused significant degradation of AI performance, compromising the consistency of AI performance over time. Molecular testing of HPV was done in addition to the cervical smear analysis but was often delayed owing to difficulties in procuring consumables.

Engaging stakeholders and tackling the treatment policy barriers proved vital. An ongoing dialogue with local health authorities and clinicians helped align diagnostic efforts with regional and national guidelines and ensured that women who were given diagnoses of precancerous or cancerous cervical lesions received histological confirmation and adequate and timely treatment. We observed that limited treatment capacity in the study areas influenced how diagnostic thresholds were set for interventions, with a focus on high grade and cancerous lesions.

AI enabled cytology allowed for rapid analysis at scale, reducing the need for pathologists on site and thereby expanding access to screening in resource limited settings. But our experience reinforces the fact that introducing innovative diagnostic methods without concurrent investment in health system integration, reagent supply, treatment availability, and community trust risks creating fragmented and ineffective care. AI can support task shifting to community health workers, but only if adequate training, supervision, and referral systems are in place.

## Implications and recommendations

*Ensure technological fit*—Prioritise tools that work offline, are robust in low resource settings, and match local epidemiology and clinical workflows.

To successfully implement and scale up AI supported diagnostic tools in resource limited settings, robust and context appropriate infrastructure is essential. Innovations should be designed for real world conditions, ensuring usability in low connectivity and resource constrained settings. Novel AI based methods are needed that can be applied and function reliably in settings with unstable or limited access to electricity. Portable obstetric ultrasonography devices designed to function offline in rural clinics have improved antenatal care access in sub-Saharan Africa.^18^ ^35^ Our work in East Africa confirmed that technologies must be adapted to local realities, making them affordable, portable, and usable without requiring advanced technical expertise. The development of these technologies should prioritise integration of locally implemented, offline diagnostic systems (known as “edge AI”) as an alternative to cloud based solutions. This would enable diagnostic tools to be used on-site, reducing dependence on internet connectivity and cloud infrastructure.^18^ ^36^

*Invest in workforce development*—Train and support healthcare workers to integrate AI tools into routine care, with clear referral and treatment protocols.

Local laboratory capacity must be strengthened through training personnel and introducing clear quality assurance protocols. Technology developers should work closely with local health providers and patients to ensure tools are practical, trusted, and responsive to local needs. Methods that previously required high level expertise and manual analysis can increasingly be automated, but there is a substantial translation gap, in high and especially in low resource settings.^6^ ^23^

Diagnostic practices that currently rely on human expert assessment, such as pathology and radiology, are eligible for automation with AI, which enables improved accuracy and task shifting.^5^ ^15^ ^23^ Results from large scale implementation studies of AI supported cytological cervical cancer screening, for example, show that manual review workload can be substantially reduced, false negatives decreased, screening coverage increased, and triage turnaround times cut from days to hours.^5^ ^36^ For cytological screening of cervical cancer, these results call for a re-evaluation of performance against other diagnostic modalities that can be hard to implement in LMICs, such as molecular HPV testing.

Regarding portable ultrasonography, AI can be used to simplify protocols and automatically interpret the images acquired by sweeping an ultrasound device across the patient’s abdomen, a procedure that can easily be taught to, for example, nurses and technicians.^18^ Screening for breast cancer can be supported by non-invasive, thermal AI based imaging, and used by primary healthcare workers as a triage tool in places where access to mammography is limited.^16^

AI based triage systems might beneficially incorporate agentic (that is, autonomously acting), multimodal AI that can prioritise high risk cases for expert review and also provide access to relevant guidelines and treatment protocols, thereby improving diagnostic efficiency and accuracy.

*Establish clear regulatory and ethical frameworks*—Support LMIC governments in developing policies that enable safe, equitable adoption of AI diagnostic solutions.

Action is needed from multiple stakeholders. Governments and health authorities have critical roles in establishing regulatory frameworks, screening policies, and sustainable financing models. Regulatory bodies must facilitate adaptive evaluation pathways that encourage both safety and innovation. Most LMICs still rely on general data protection statutes and medical device laws,^37^ but promising national initiatives to regulate medical use of AI are increasingly being developed in LMICs,^38^ and international guidelines for how to establish regulatory frameworks have been published.^39^ ^40^

*Create sustainable financing models*—Encourage pooled procurement, insurance coverage, and public-private partnerships that cover full implementation costs, including maintenance and training.

To accelerate adoption and integration, we recommend that AI supported diagnostic methods are embedded into national disease control programmes, establishing reimbursement mechanisms for digital screening tools and fostering public-private partnerships to scale up innovation responsibly. With such measures, AI supported cytology and other AI based diagnostic methods could become a key component of equitable cancer prevention and women’s health related conditions in low resource settings.

*Engage communities in design and delivery*—Co-design tools with end users, including patients and providers, to ensure cultural relevance and build trust.

Raising awareness of diseases that can be diagnosed with novel AI based methods, such as cervical and breast cancers, is crucial.^22^ Around one third of all countries globally (34%) have implemented an m-health initiative in the past two years that supported the achievement of health objectives such as awareness of non-communicable diseases.^41^

Successful implementation depends on human oversight and adherence to existing regulations, with continuous monitoring. A multifaceted approach is required for large scale roll-out: investing in basic infrastructure, training healthcare workers, forming collaborative networks of experts, ensuring regulatory clarity, and engaging communities. Partnerships with governments, non-governmental organisations, and technology companies can drive policy development, secure funding, and support effective implementation. Technology should be adapted to existing infrastructure and integrated into healthcare pathways that link improved access to diagnostic services with treatment. Pilot regional programmes should be expanded when proved effective, and efforts to build public awareness will help foster trust and uptake.

## Conclusions

AI supported diagnostic methods have the potential to dramatically improve health outcomes for women in LMICs by enabling earlier and more accurate detection of a range of important medical conditions. But this potential can only be realised through deliberate investment in implementation systems, policy frameworks, and community engagement. Cervical cancer screening is an important example, but the lessons learnt are widely applicable across women’s health. A system strengthening approach to scale up AI diagnostic tools is essential to close persistent gaps in access and to realise the promise of digital health equity.

Our experience with AI based cervical cancer screening in East Africa shows that digital diagnostic methods can be implemented at the primary healthcare level in resource limited settings, provided they are carefully adapted to local conditions and embedded in functional health systems. Although our insights may not be universally applicable and are limited by the context specific nature of the studies, we think they provide valuable lessons for the wider discussion on how to integrate AI supported diagnostic methods. Despite the significant potential, the diagnostic accuracy, efficiency, and technology are not enough. Success depends on investing in infrastructure, building human and institutional capacity, and ensuring adequate and timely treatment availability. Strong stakeholder engagement and ethical implementation are essential to ensure that diagnostic advances translate into real health gains.

**Key messages**

AI supported diagnostic methods can expand access to screening for cervical cancer and other women’s health conditions in low resource settings
Impact depends on integration with the local health systems, not just use of the AI technology
Supply chains, trained staff, and functional referral systems for appropriate care remain major barriers
Strong regulation, ethics, awareness, and financing are essential for scale-up and equitable access to AI supported diagnostic tools
More innovation is needed to simplify the diagnostic pathways and methods and make them less dependent on infrastructure, consumables, and skills of the user

## Notes

Contributors and sources: NL is a medical doctor and a professor in AI based diagnostics at the Department of Women’s and Children’s Health at Uppsala University, Sweden, and a senior researcher at the Institute for Molecular Medicine Finland (FIMM) at the University of Helsinki, Finland. DN is a medical doctor and manages the AI based screening projects for cervical cancer in Tanzania. AM is a medical doctor and a professor of international child health at the Department of Women’s and Children’s Health at Uppsala University, Sweden. HK is chief executive officer at Kinondo Hospital, Kenya, and oversees the clinical studies in Kinondo, Kenya. BN is a medical doctor and professor of parasitology at Muhimbili University of Health and Allied Sciences (MUHAS). JL is a medical doctor and professor of medical technology at Karolinska Institutet, Sweden, and a research director at FIMM, University of Helsinki, Finland.

This article is part of a collection developed in partnership with the Gates Foundation. Open access fees were funded by the Gates Foundation. *The BMJ* commissioned, peer reviewed, edited, and made the decision to publish. Smruti Patel and Jocalyn Clark were the lead editors for *The BMJ*.
